# Oncology nursing in the Global South during COVID-19

**DOI:** 10.3332/ecancer.2021.1329

**Published:** 2021-12-09

**Authors:** Julia Challinor, Maria Fernanda Olarte Sierra, Kathryn Burns, Annie Young

**Affiliations:** 1 University of California San Francisco, 2 Koret Way, San Francisco, CA 94143, USA; 2 Independent Medical Anthropology, San Sebastián, Spain; 3 Qualitative Research, Budapest, Hungary; 4 Warwick Medical School, University of Warwick, Coventry CV4 7AL, UK; a https://orcid.org/0000-0002-5008-8501; b https://orcid.org/0000-0002-6537-7138; c https://orcid.org/0000-0002-2695-1088; d https://orcid.org/0000-0001-6611-6653

**Keywords:** COVID-19, oncology nursing, psychosocial support systems, mental health

## Abstract

In mid-2020, a call was made to oncology nurses in the Global South to share their experiences managing patient care during the coronavirus disease 2019 (COVID-19) pandemic. Eighteen submissions were received from 16 countries across Latin America, Africa, Europe and Asia. Three were research-based and 15 were personal narratives on the psychosocial impact of COVID-19 on the nurses, colleagues, patients and families. Three narratives were from oncology nurses working with cancer-related non-governmental organisations locally or, in one case, internationally. A simultaneous literature search for publications (including grey literature) was performed to identify themes of COVID-19’s impact in these 16 countries and specifically on oncology nurses and patients/families. Four themes were identified: a) interruptions to care; b) support/resource shortages; c) psychosocial impact on nurses and patients and d) staffing and nursing role impacts. The three research-based studies describe oncology nursing in-depth efforts to explore the impact of COVID-19. Findings in the 15 narratives are briefly presented according to the four themes identified in the literature. Due to the severe shortage of physician adult and paediatric oncology specialists, oncology nurses in the Global South often shoulder much of the care for patients with cancer and even more so during COVID-19 with attendant oncology nursing shortages due to reassignment to COVID-19 units. It is important to hear from these critical members of the oncology nursing workforce who often lack the time, resources or training to publish in peer-reviewed journals in English, particularly in the middle of a pandemic. Giving voice to these nurses documents the reality of their work and ability to continue to provide care despite the chaos and rapidly changing guidelines and government action. Lessons learned by these nurses to improve mental health and psychosocial support of the nurses as well as their patients/families will be essential for the next global pandemic.

## Background

Historical global infectious diseases (e.g. tuberculosis, malaria, HIV) are increasingly being brought under control, even in underserved communities. ‘Overall, deaths caused by AIDS, TB and malaria each year have been reduced by nearly 50% since the peak of the epidemics in countries where the Global Fund invests’ [1]. However, cancer is emerging even in underserved communities as a public health threat and recognised by the World Health Organization (WHO) in a resolution in 2017 [2].

Across the world there are disparities in cancer control and treatment between and within countries and regions. In many countries in the Global South^1^, complex cancer treatments are not available due to the lack of radiation therapy and oncology surgeons, inconsistent access to essential medicines and chemotherapy and fragile healthcare infrastructures.

Between-country comparisons primarily reflect inequalities in the overall pattern of exposure to risk factors, as well as in the availability of and access to the relevant health services. Comparisons within a country reveal how inequalities between groups of fellow citizens affect their cancer outcomes, at least partially reflecting differences in access to the available health services. International comparisons can guide national cancer control priorities, whereas the subtleties of cancer patterns within countries may reveal important indicators for targeted cancer control measures [4, p.3]. For example, in the USA, although the incidence rate of breast cancer is the same across racial/ethnic groups, Black women have higher morbidity, and, for renal cancer, American Indians/Alaska Natives have higher morbidity than any other group [5].

Nurses caring for patients with cancer across the world may or may not have received training to do so. Depending on their setting, they may be required to prepare chemotherapy as well as administer it. They may have to manage end-of-life care for patients who succumb to their disease both in the hospital and at home. Nurses are uniquely positioned within their communities to advocate for cancer screening and healthy lifestyles to avoid cancer risk. They are also resources for families of patients with cancer and survivors to educate communities to diminish stigma and model support for these community members. In other words, nurses are fundamental to cancer surveillance and care as well as life after treatment.

The coronavirus disease 2019 (COVID-19) pandemic has had a devastating impact on cancer treatment and care. The pandemic began in China in late 2019, but rapidly moved across Asia and to the West affecting Europe, then the USA, and Latin America and finally Africa. Across the world, patients with cancer were afraid to go to the hospital for treatment and cancer screening rates dropped precipitously as healthcare systems were overwhelmed by the critical needs of patients with COVID-19. Oncology nurses were rotated to COVID-19 wards, and vulnerable nurses (such as those pregnant or with underlying chronic illnesses) were often placed on leave. Scheduled oncology surgeries were cancelled and all but the most critical chemotherapies were delayed.

Nurses caring for patients with cancer still had to manage personal protective equipment (PPE) while handling hazardous drugs (e.g. chemotherapy), but hospitals faced serious shortage of PPE and much of what was available was diverted for use by healthcare and other personnel managing patients with COVID-19. Many nurses also feared for their families’ well-being as they had to work in the facilities where COVID-19 patients were being attended to. Thus, many moved away from their homes or had their relatives relocated to decrease the risk of bringing the virus home.

This collection of articles addresses the psychosocial consequences of providing nursing care to patients with cancer during a pandemic. We asked oncology nurses across the globe to reflect on their mental health and psychosocial experiences in early days of the COVID-19 pandemic. We do not divide the stories by their authors’ country-income status. Rather, we consider the UN Development Programme human development index (HDI) (health dimension, education dimension and standard of living (gross national income per capita)) [6] to cluster these contributions in addition to other demographic and cancer-related indicators. This decision allows us to consider the in-country as well as across country disparities of cancer care and health care in general.

Our research aim was to document the mental health and psychosocial lived experiences of nurses caring for patients with cancer in countries with low HDI during a pandemic. We sought to make visible the story that was not being told in the mainstream media, overwhelmed with reporting on the details of COVID-19 care and consequences. This is a collection of the work of oncology nursing practice in the shadow of COVID-19 and how the nurses and local foundations supporting patients with cancer had to pivot and adapt to maintain good practices and personal safety under unknown circumstances and risk.

## Methods

### Desk review

To contextualise the articles submitted by oncology nurses across the Global South, we began by compiling data and statistics related to COVID-19’s spread across our focus countries, including the date COVID-19 was first identified in each country, and a timeline to the end of 2020. We also identified data on country HDI, total population, total cancer cases in 2018 (most recent year available) and % of gross domestic product (GDP) spent on healthcare. HDI is calculated according to three indicators: long healthy life, knowledge, a decent standard of living. The health dimension is assessed by life expectancy at birth, the education dimension is measured by a mean of years of schooling for adults aged 25 years and more and expected years of schooling for children of school entering age. The standard of living dimension is measured by gross national income per capita [6].

We then conducted a focused literature search for academic literature published from fall 2019–December 2020 related to oncology and the impact of COVID-19 across the 16 countries included in this issue. We searched for articles published in English and Spanish (using Google scholar) with the following search terms: COVID-19 and oncology + [country); ‘Impact of COVID-19 on cancer treatment in [country]’; cancer care during COVID-19 + [country]; COVID and oncology + [country] + nursing; COVID-19 and oncology patient [/family] + [country] in 2020. A small number of grey literature articles were also identified through a Google search. Articles were then further narrowed down based on their relevance to oncology nursing. The final focused literature review, presented in the results section below, includes those publications addressing oncology nursing and COVID-19’s psychosocial and practice-related impacts including in the 16 focus countries.

### Recruitment

The senior and co-editor oncology nurses recruited colleagues who were oncology nurses from the Global South to participate in this special issue by email. Considering the serious impact on nursing practice in the first months of the pandemic, long-standing professional relationships allowed for inquiries of potential collaboration without pressure or coercion. In countries where we had no personal oncology nursing contacts, we solicited known adult/paediatric oncologists to determine if nurses working in their units would be interested. Oncology nurses working in and with non-governmental organisations (NGOs) in (or in one case supporting) paediatric oncology in the Global South were also recruited to consider writing about the impact of COVID-19 on their psychosocial support of nurses, families and patients with cancer.

### Guiding documents

Oncology nurse authors were given the framework of the WHO ‘Mental health and psychosocial considerations during the COVID-19 outbreak’ [7] as a starting point for their contributions. Psychosocial standards of care were also shared (e.g. Mattie Miracle Foundation for paediatric oncology) [8] and the International Psycho-oncology Society standards for quality cancer care [9]. It was expected that the approach to reflecting on and explaining the psychosocial impact of COVID-19 would vary according to region and practice. Therefore, each author was encouraged to write their story in a format that was appropriate to their setting and pandemic experience, and hence the lack of uniformity in the papers herein. This special issue offers the unique possibility to appreciate the diverse, yet often similar, approaches oncology nurses have taken to manage and make sense of the psychosocial impact of COVID-19 on cancer care in their settings.

### Language

Spanish-speaking nurses (non-English language speakers) from Latin America were interviewed by an experienced Colombian medical anthropologist by phone and their interviews were analysed in their original language. The most salient elements were translated to English. The Iranian oncology nurse’s contribution in Farsi was translated by an experienced Iranian sociologist. The Iraqi oncology nurse’s contribution was written in Arabic and translated to verbatim English by an Iraqi oncologist who is fluent in English.

## Results

**Figure 1.**
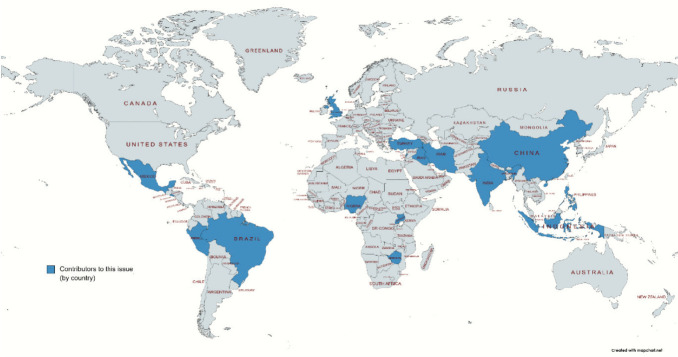
Map of contributing nurse authors’ countries.

**Figure 2.**
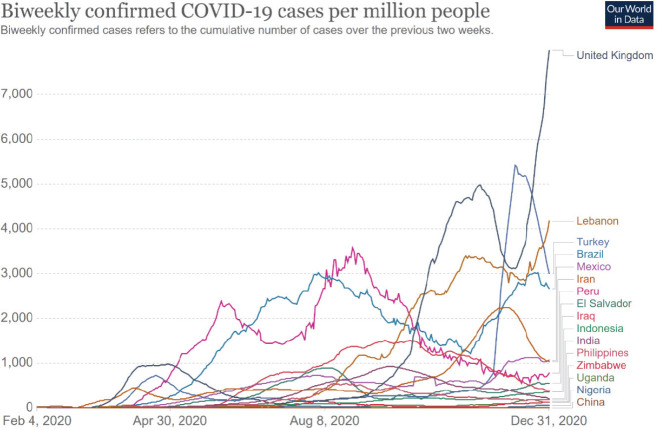
COVID-19 cases in 2020 by countries of nurse contributors.

**Source: Dong E, Du H, and Gardner L (2020) An interactive web-based dashboard to track COVID-19 in real time** *Lancet Infect Dis* **20(5) 533–534**

Eighteen submissions were received from 16 countries across Latin America, Africa, Europe and Asia (see Figure 1). Three were research-based and 15 were personal narratives on the psychosocial impact of COVID-19 on the nurses, colleagues, patients and families. Three narratives were from oncology nurses working with cancer-related NGOs locally or in one case, internationally (see Table 1). Table 2 presents the HDI, total population, total cancer cases in 2018 and percent GDP spent on healthcare for each country. The number of COVID-19 cases for each country from February–December 2020, with peaks, is displayed in Figure 2.

### Literature review results

Cancer treatment is carefully controlled by protocols or treatment regimens with time-sensitive scheduling based on clinical trial data, evidence and best practices. The complexity of delivering cancer treatment with the high risk of serious side effects and long-term negative health consequences as well as the risk of relapse and/or secondary cancers raises the stakes for disruptions in care, resources, staffing. Oncology nurse and patient/family psychosocial health is essential to continuous care as planned. Our literature review of publications related to COVID-19’s impact on oncology nursing revealed four themes: a) interruptions to care; b) support/resource shortages; c) psychosocial impact to nurses and patients and d) staffing and nursing role. These universal themes on oncology nursing and early COVID-19 pandemic consequences across WHO regions are addressed below with one or two examples for each from the available literature in 2020.

**Interruptions to care**. Cancer treatments for patients on therapy were significantly scaled back due to restricted public transportation and lack of accommodation for those travelling from rural areas (e.g. India [10]; Indonesia [11]), patient fears about going to a hospital setting with risk of COVID-19 (e.g. India [12]), cancelled non-urgent procedures (e.g. surgery, screenings) (e.g. Nigeria [13, 14]); lack of consensus on best cancer care practices during COVID-19 in some areas (as discussed in letters to the editor in Brazil [15, 16], among other factors).

**Support/resource shortages and increased financial crises.** COVID-19 also precipitated new limitations on access to medications and other treatment necessities in the wake of national financial and health care system disruptions (Lebanon post-explosion [17], Zimbabwe post-economic crisis [18], Indonesia post-massive healthcare funding cuts [10]); shortages in medications due to panic stockpiling (Iraq [19], Uganda [20]); unexplained disruptions in supply chain (Mexico [21, 22], Zimbabwe [23]). Overall increasing costs of care were due to PPE and COVID-19 tests being in high demand and available resources being diverted to COVID-19 care wards (Nigeria [24], Peru [25]). Patients and families had additional out-of-pocket costs they could not meet (India [10]) and telehealth alternatives were not possible for patients in rural areas or without access to the Internet or consistent phone access (e.g. Peru [25]).

**Psychosocial impact to nurses and patients.** Literature from across the focus countries in this issue emphasised the psychosocial impact of COVID-19 on both nurses and patients and families. Nurses reported levels experiencing increased fatigue, anxiety, insomnia, depression and burnout (e.g. China [26]), which posed risks to quality of care and nurse and patient well-being. Meanwhile, patients and families experienced heightened distress, anxiety and care system confusion (e.g. Iran [27]; Nigeria [28]), which were exacerbated by decreased psychosocial support (Lebanon Cancer Control Survey [29]) and increased isolation (India [30]), and in one case, due to the suspension of peer group and psychological sessions (India [31]) and isolation from families due to risk of infection and high rates of complications and mortality in oncology patients who contracted COVID due to the interplay of chemotherapy and immunosuppression (e.g. Brazil [32]).

**Staffing and nursing.** An additional impact of COVID-19 was decreased staff specialised in cancer management. Nurses in paediatric and adult oncology units were deployed to other COVID-19 units due to high demand for skilled hands (e.g. Iran [33]) and leaves of absence increased due to widespread COVID-19 infection of healthcare workers (e.g. Zimbabwe [23]). Stigma attached to having COVID-19 and the lack of hospital accountability related to working in healthcare and travel, lead to patients in some areas lying about travel and exposure and subsequently infecting health professionals and other administrative staff (Philippines [34]). These reasons, combined with nursing strikes (Zimbabwe [23]) and healthcare worker resignations (Peru [25]) meant that in many places the delivery of services such as chemotherapy and radiotherapy treatments was impossible (India [30]; Turkey [35]) and hospitals and clinics were ‘manned by student nurses, junior doctors, and other staff who have no choice as they are still under training’, which contributed to higher morbidity and mortality rates (Zimbabwe [23]).

### Conclusion

Across the Global South, the literature documenting the four identified themes make clear that cancer treatments were sidelined by healthcare systems worldwide as they struggled to manage the COVID-19 pandemic. These themes are reflected and detailed in the narratives presented here from the nurses in the 16 focus countries in the Global South during the first wave of the pandemic in early 2020. We present how the nurses’ experiences during the pandemic altered and disrupted their lives at a personal and professional level and the lives of their patients and families. Through their voices, seldom featured in the media, we have a unique vantage point to come closer and truly understand the challenges, practices and possibilities of oncology nurses brought about by the COVID-19 pandemic. That is, to attend to what the pandemic has meant for nurses caring for patients with cancer and their families daily and in their local contexts. In this way, the reader will hear the similarities and nuances of the impacts of COVID-19 as it marched across the Global South.

## Invited papers

Three of the 18 papers were based on quantitative research. The format of each of the 15 personal narratives is distinct but gives voice to the nurses’ insight and on-the-ground lived experiences. These descriptions offer a snapshot of the immediate dramatic negative pandemic impact on patients with cancer and their families as well as the fallout on nursing practice and nurses’ mental health and ability to provide appropriate care.

### Quantitative research

Teixeira and co-authors present an analytical and qualitative reflection on the psychosocial impact of COVID-19 on oncology nurses, based on literature published in Portuguese and English (mainly Brazilian), and governmental and non-governmental website reports. In addition, the authors reflect on their personal experiences during early days of Severe Acute Respiratory Syndrome Coronavirus 2 (SARS-CoV-2). They frame the oncology nurses’ experiences within the Brazilian public health system – Brazilian Unified Health System (SUS) – and how patients with cancer access treatment. They note a significant decrease in cancer-related surgeries and biopsies were documented in the first 8 weeks of the pandemic, and in a survey by a large oncology institution, just under half of the respondents mentioned that the pandemic had impacted their treatment. The authors highlighted the mental health impact of COVID-19 on patients and oncology nurses throughout the country. One example of action to address this was a description of the Brazilian Federal Council of Nursing’s ‘Solidary Nursing Program’ effort to establish safety guidelines and 24 hour/7 day a week online mental health support. Other interventions to improve nurses’ resilience were documented, e.g., national oncology societies’ collaboration with health communication, pharmaceutical and medical device companies to conduct webinars and educational on-demand resources to address healthcare worker questions and concerns.

In India, Jagdish and colleagues from Tata Memorial Hospital in Mumbai, describe a nursing and psychology investigation of the impact of COVID-19 on 50 adult oncology patients with COVID-19 recovering at home. The patients were interviewed about physical, emotional, social using an investigator-designed tool validated by seven local experts. They describe the high-level emotional problems of patients with solid tumours compared to those with haematological cancer and the lower emotional impact felt by patients receiving radiotherapy compared to other treatment modalities. The authors also note the uncertainty of patient caregivers and difficulty fulfilling their role with social distancing and masking.

Xu and collaborators in Hong Kong, submitted a scoping review of Chinese- and English-language oncology nursing literature about oncology psychological care and COVID-19 in mainland China published from December 2019 to August 2020. The findings of three English-language and 16 Chinese-language eligible articles indicated that geography and specific cancer diagnoses had distinct impacts. The lack of structured, effective psychological patient support was noted and oncology patient stress-reduction interventions are highlighted to mitigate early depression and anxiety as well as long-term consequences.

### Narratives of oncology nurses and COVID-19

Across 11 countries (China (2 narratives), Lebanon, Indonesia, Turkey (3 narratives), Iran, Iraq, Nigeria, Uganda, Mexico, El Salvador, Peru, Philippines, Zimbabwe) and multiple sites, oncology nurses wrote narratives reflecting the literature on the impact of COVID-19. Each nursing narrative is framed by the local COVID-19 situation, hospital and public health authority response, and the oncology nursing care setting. The nurses’ descriptions of the extent of the psychosocial impact of the pandemic on the nurses themselves, as well as their patients and families are presented according to the themes described in the literature review mentioned above. These narratives provide a vivid picture of how caring for oncology patients amidst a pandemic looked like. Which were the work, family and social consequences that nurses and their patients faced.

The decision for inviting oncology nurses’ narratives is based on the fact that we sought a closer look at their rich experiences. To gain a comprehensive understanding, the methodology required a small sample of persons and interviews (depending on the circumstances). As Crouch and McKenzie [36] highlight, qualitative research validity does not depend on the size of the sample. Validation stems from the quality and depth of the data. Gross generalisations in this kind of research are not desirable since the focus is on individual experience. However, given that people live in a socio-cultural context that informs and permeates their lives, a qualitative approach gives space to understand why and how social phenomena occur [37], and not only the frequency in which it does. In this sense, the experiences nurses refer to here can, indeed, be considered characteristic of their context.

### Interruptions to care

In Iran, the oncology nurse was transferred to care in a pulmonary ward, but noted, ‘there were no pulmonary disease patients around anymore but Corona patients. All the beds were emptied to receive Corona patients’. From Ankara, Turkey, an oncology nurse reported, ‘except for the planned chemotherapy medications, all other elective appointments were postponed’. Public transport in Uganda stopped completely during lockdown thus affecting families who abandoned care since they could not get to the hospital. Overcrowding occurred in the hospital due to families not being able to return home. Nurses were unable to reach the hospital, thus those living close by had to take extra shifts.

Nurses reported families’ fears about going to the hospital at all because of possible contagion; in Istanbul, Turkey, ‘many cancer patients did not want to come to the hospital and postponed their controls’. In Iraq, the hospital was in a temporary structure due to the war in 2017, and lacked all radiotherapy services. Since movement in and out of the city due to COVID-19 was restricted, no patient could receive radiotherapy normally delivered in Baghdad. In Lebanon, following the explosion in 2020, an already downsized healthcare system in financial crisis cancelled cancer awareness programmes and free mammograms. Closed city borders and fear of contagion were also factors in decreased paediatric oncology visits in Istanbul, Turkey.

### Support/resource shortages and increased financial crises

Shortages of PPE were of specific concern to the oncology nurses and highlighted by nurses in Iraq, El Salvador, Uganda and Indonesia. Patient and paediatric family financial crises impacted ability to attend to hospital appointments due to public transportation shutdowns (Uganda, China), and inability to pay for hospital tests or procedures or accommodation (Nigeria, Uganda and Iraq), or pay for a COVID-19 test (Indonesia).

Ndagire and Nabakoosa from Uganda, mentioned oncology nurses’ great fear of contracting COVID-19 and the lack of testing. Shortages of PPE and inconsistent use by families of hospitalised patients add to their concerns for personal safety as well as their families. In Nigeria, Anarado and colleagues described oncology nurses and families as ‘swinging from helpfulness to helplessness to hopelessness’. Nurses were providing families with out-of-pocket financial support, prayer, counselling and advocacy. They also noted the need for hospital attention to improve PPE supplies, counselling for the nurses to address their mental health and capacity building for supporting patients with cancer and their families.

Nurses in Ankara, Turkey, mentioned family unemployment causing financial difficulties.

### Staffing and nursing

An oncology nurse from Mexico shared, ‘If I stop to think about all that is going on, I would crumble. We would crumble. I need to be okay so I can contain others who are in a more difficult condition than I am. I must do what I have to do and more. My duty is to care for the patients. Nothing else matters.’

A second contribution from Istanbul, Turkey, by an adult oncology nurse mentioned difficulties in normal nursing communication with families of patients with cancer due to nervousness: ‘Many nurses are caring for patients with cancer, perhaps for the first time, and have had to manage their patients’ concerns related to cancer treatment and feelings of hopelessness about their COVID-19 infection’. Here too, oncology nurses experienced isolated living situations when they decided to separate from their families to prevent potential infection with COVID-19 as they continued to provide hospital-based care.

In Shanghai, the author, He and collaborators share their perspective of paediatric oncology nursing during the pandemic, including trying to manage misinformation and rumours by redirecting patients and families to rely on trustworthy sources like health professionals. Patient communication was also challenging. In Indonesia, an oncology nurse noted a reduction in therapeutic communication from nurses to patients due to the nurses’ increased workload and COVID-19-risk-related distress.

Stigma related to having had COVID-19 was noted by oncology nurses in Turkey and Indonesia. The Indonesian nurse stated, ‘The COVID-19 stigma in the Indonesian society is strong. Some nurses had been expelled from their rented houses because the owners were afraid that they might spread the disease’. In Ankara, the paediatric oncology nurses mentioned they were caring for a child whose parent had contracted COVID-19 and was stigmatised when she returned to another hospital with her child. She was grateful that she did not experience this in the nurse’s unit. In Lebanon, the Order of Nurses began a campaign to address healthcare worker stigma with ‘slogans such as: Nurses are the army of the health care system’.

Interviews with oncology nurses in Peru, Mexico and El Salvador highlighted emotional responses to the inability to hug and kiss colleagues and their paediatric patients and parents, which was an abrupt significant culturally unacceptable change in their nursing practice. In the Global South, with limited high-tech biomedical equipment and therapies, it is often the human acts of physical support that are critical in oncology nursing and care.

Finally, several nurses described families/parents not adhering to infection control teaching/protocols, despite targeted and repeated teaching on this topic to reduce the risk of COVID-19 infections. This was noted in Ankara, Turkey; Iraq and Uganda. The contributing author from Iraq, an oncology nurse, noted, ‘Patients are still coming with their relatives, and many were not adhering to the preventive pandemic instructions’.

From Lebanon, nurse Doumit poignantly presents the fundamental role that the Lebanese Order of Nurses played in ensuring nurses who had been exposed to COVID-19 no longer had to self-quarantine at their own expense and arranged for free accommodation. In Uganda, existing nursing shortages were exacerbated by transportation difficulties, exposures to patients with COVID-19 and secondment to other units. Remaining oncology nurses had to work longer hours and additional shifts to meet the demand of even reduced numbers of patients with cancer.

### Psychosocial impact to nurses and patients

In Istanbul, Turkey, a paediatric oncology nurse mentioned, ‘I was afraid of infecting my daughter and parents who had moved to my house to take care of her, because schools were closed and I had to work in the hospital. At home, I stayed in another room, it has been months that I have not hugged my daughter’.

Afiyanti and Milanti from Indonesia detailed the nurses’ need to maintain distance between people, limit the number of hospital visitors (or no visitors at all) and the impossibility of hugging and touching, which was interpreted by patients and their families as rudeness and sometimes neglect by the oncology nurses. The authors reflected on how oncology nurses had to make sure that the pandemic safety measures were strictly followed by all. The oncology nurses report praying more frequently and more devotedly for their patients, which they describe as a ‘core value’ of Indonesians.

The nurses from Africa highlighted the role of religion and the need of further patient psychosocial counselling since their patients lacked traditional spiritual support due to community lockdowns, which included religious centres. Here too, oncology nurses engaged in extra praying for their patients as the fear of relapse and death filled nurses’ descriptions of the current pandemic situation. However, spiritual support for families was not always positive, as highlighted by a Nigerian oncology nurse,

The most commonly coping strategy offered to patients and caregivers by nurses was prayer and counselling to commit all to God. Nigerians are very religious and may be almost ‘gullible’ to any pastors preaching. Some dubious pastors may exploit nurses and patients or take away their last coin, with the promise of praying for them.

Three contributions from Turkey illustrate the diversity of the psychosocial impact of the pandemic on oncology nursing. In the first paper from Ankara, two nurses share their personal stories of getting their family to safety first, in order to work during the pandemic. The Chief Nurse then describes the burden of her leadership responsibility in unknown and previously unencountered circumstances. Managing fevers of children with neutropenia and new protocols to screen for COVID-19 as well as shortages of PPE required for chemotherapy handling were two examples of daily stressors. A staff nurse described her struggle to regulate her concerns and practices to guard against COVID-19 infection while maintaining an equilibrium between anxiety and reasonable caution.

From Iran, an oncology nurse shared, ‘Two nurse colleagues and I plus two medical assistants and one of the administrators at the nursing station were awaiting the Corona patients to arrive and get admitted. The patients used to describe us: “people from the space/aliens!” with our strange looking gowns. We were sitting and in a strange silence we were thinking of leaving, resignation, fear of death, and …’

In Shanghai, China, most family and patient psychosocial distress was related to at least one or two generally tangible needs including: quarantine, transportation, masks, blood products, etc. Oncology nurses seconded to Wuhan, China described mindfulness training, tai chi exercise and journaling to patients who were hospitalised and experiencing severe psychosocial distress.

One psychosocial sub-topic which arose in the nursing narratives that was not mentioned in the literature was nurse vulnerability. In Ankara, Turkey, oncology nurses reported having to limit their social relationships for safety and eat alone in the hospital and at home. They also mentioned psychological exhaustion. In El Salvador, the nurse stated, ‘we are all very scared. Many colleagues from the hospital have lost relatives, and also a lot of healthcare personnel have died (…). My fear is to get the virus and then get complications and die’. An oncology nurse in Mexico reported feeling as though it was inevitable that she would contract COVID-19, and her greatest fear was dying alone without seeing her daughters and leaving them alone.

In Lebanon, an oncology nurse reported being affected by social bullying of nurses even by friends and family members because they continued to attend to patients in the hospital. The rising patient ratio for nursing care and longer shift hours are leading to nurse burnout in Uganda due to COVID-19. In Iraq, an oncology nurse reported his, ‘dread of the disease and precautions in managing patients (during COVID-19) has increased the stress of work’.

### Oncology nurses working in or with a local or international NGO

Two oncology nurses contributed narratives about their work in local cancer-related NGOs in the Philippines (Cancer Warriors Foundation, Inc. (CWFI)) and in Zimbabwe (Island Hospice and Healthcare) during the pandemic. An oncology nurse and counsellor from the UK shared their experience working in an international NGO (World Child Cancer) supporting multiple countries in the Global South. Unlike the Global North, where patients generally have insurance and NGO role is normally to provide social support and political advocacy, NGOs in the Global South often routinely provide medications (including chemotherapy), nursing training, financing for laboratory and other testing and family accommodation during a patient’s treatment (as well as food). Therefore, when the pandemic began, travel restrictions were put in place and charity donations plunged worldwide [38], and particularly in the UK where World Child Cancer is located.

In the Philippines, oncology nurse Villarta noted that CWFI initiated phone call psychosocial support to ‘post greetings, informational materials, inspirational quotes, photos and a lot more, that would make the kids, their caregivers and other family members feel well, happy, hopeful’. Patients and parents in extreme psychological distress were directed to professional online support services. They also directed families to services for exercise including qi gong. Due to quarantine shutdowns, families of children/adolescents with cancer suffered from a lack of employment and required basics such as food supplementation provided by CWFI to ensure reasonable nutrition during cancer care and survivorship.

In Zimbabwe, oncology nurse Tsikai, a palliative care consultant describes the high level of patient counselling required, both in person and with telemedicine with newly trained community volunteers, due to the interruptions of cancer care, double stigma of cancer and COVID-19 and generalised fear of death.

World Child Cancer UK provides support for 13 programmes in the Global South as well as individual hospitals in multiple countries. The contribution notes the stigma oncology nurses faced from working in hospitals as well as the longer hours due to staff shortages in several partner sites. In person training support for oncology nurses was halted due to travel restrictions and for safety reasons, thus the NGO had to pivot to online telephone and Internet communications support. Posters on healthcare worker resilience strategies were created in multiple languages to acknowledge the psychosocial and mental health stress of the local workforce in the face of great patient and family distress across sites.

## Discussion

The research-based work and narratives presented here demonstrate that many oncology nurses across the Global South were at the breaking point in mid-2020. Some had died, others had quit and many were burned out. Despite these circumstances, the majority remained in place and continued to deliver oncology care, even though patients had shown reluctance or refused to come to hospital out of fear of contagion. Patients arriving with more advanced disease at diagnosis and cancer treatments that had been delayed or cancelled have led to relapses, or a higher risk of relapse, despite patients’ intensive treatment at diagnosis.

Overwhelming second and third waves of COVID-19 in the Global South, such as in India and Latin America, highlight the fragile infrastructure to manage a large-scale pandemic and lack of access to vaccines, which also puts the spotlight on global health care disparities and cancer care in particular since it requires complex treatment. Several countries mentioned in this paper had significant economic or political crises (financial collapse, explosion or conflict), so the healthcare infrastructure was already under stress.

Cancer care is complex at the best of times and requires multimodal treatments and consistent access to chemotherapy, surgery and radiotherapy. The pandemic has threatened and, in some cases, interrupted access to all the aforementioned treatments and screening. It is now apparent that the decrease in screening activities and delays in scheduled treatment due to chemotherapy being out of stock and catastrophic routine hospital infrastructure collapse due to COVID-19, have resulted in overwhelming numbers of sick patients needing intensive care by staff already affected by shortages and lack of intensive care beds.

In general, oncology nurses are accustomed to caring for patients at the end of life in their practice, and they have experience managing their feelings and sadness. However, it is particularly devastating to have worked so hard to cure a patient with cancer, only to see them die of COVID-19. There is no chemotherapy for COVID-19, and limited treatment options. We take heart from the resilience, ingenuity and strength of the nurses in the Global South, and NGOs who support patients with cancer who document herein their valiant efforts to persevere and demonstrate their solidarity with patients with cancer in the face of health and social crisis due to the pandemic.

Given COVID-19’s indiscriminate nature, that has exposed vulnerabilities of healthcare systems, governments and even public health authorities and experts, even in the Global North, documenting oncology nurses’ personal and professional psychosocial experiences during the pandemic acknowledges their resilience and fortitude. The non-stop hands-on care by frontline oncology nurses and their support of families of newly diagnosed patients with cancer, those already on treatment that is now sometimes delayed causing significant distress and anxiety, and survivors who rightly worry about their health, is testimony to the invaluable workforce that oncology nursing represents. Cancer does not pause for a pandemic – and oncology nurses do not pause in their care, despite the added personal and professional burden and psychosocial consequences.

### Recommendations for the future

Preparedness and robust structured and continual mental health support and workplace environmental safety for oncology nurses will be paramount for future pandemics. As Datta from Kolkata, India and colleagues state,

Nourishing the mental health of staff working in a cancer center is always critical for a healthy cancer care ecosystem, but this is all the more important during the time of crisis. In LMICs (low- and middle-income countries), cancer care workers face several unique challenges in addition to the routine challenges of working during a pandemic. The lessons learned during this pandemic will be helpful for us and other institutions in LMICs to prepare for the challenges of this and future pandemics, especially with regard to preserving the mental health of health care staff [39, p.1492].

An oncology nurse in this series from Nigeria sums up needs for the future as also noted in the literature [40, 41].

Nurses and other health workers need to be motivated and encouraged by providing them materials to work; nurses need as a matter of urgency intermittent work-related counselling as COVID-19 continues to strengthen their resilience and coping in addition to an adequate supply of PPE. ‘Tele-nursing’ will support patient care when introduced and lastly, nurses in cancer care need advanced oncology education during COVID-19 to be able to deliver expert psychosocial oncology nursing care, often a neglected component of care [37, 38].

Given the serious negative psychological and emotional impact of COVID-19 on both oncology nurses and patients and families, hearing the voices and lived experiences of these nurses is critical to appreciate the scope of the consequences of this pandemic in the early days. Appropriate policy and pandemic mitigation and recovery responses in oncology care must consider the on-going experiences and continual lessons learnt from the largest single workforce – nurses.

### Strengths and limitations

This collection of articles is one of the earliest documentations of the lived experience of oncology nurses on the frontline across the Global South. These contributions provide guidance for instituting policies and practices in the future to prevent the devastating outcomes that have now caused increased nursing shortages and distress in the largest single healthcare workforce globally. As the burden of cancer increases across the Global South, the reliance on a specialised oncology nursing workforce is critical. To retain an effective oncology nursing workforce, mental health and psychosocial well-being must be addressed and preserved. The narratives within this issue reveal the reality on the ground was overlooked in initial publications focusing on decreased cancer screening, delayed surgeries and radiotherapy, shortages of protective equipment and chemotherapy and patient survival. The breadth of the oncology nurses’ reflections includes a wide range of immediate concerns during the early days of what became an enduring pandemic. The real psychosocial impact and mental distress of oncology nurses caught between professional nursing practice uncertainty and personal safety as well as that of their family are well-documented here. Another strength of the articles is that they cover both paediatric and adult oncology nursing as well as the inclusion of articles from non-English speakers. This was a deliberate decision to hear from oncology nurses who do not often have a chance to be heard in professional English-language publications.

Limitations of this collection of articles include the inability of extending a global call for contributions given the extreme situation of the pandemic. Oncology nurses were rotated to COVID-19 wards and vulnerable nurses were sent home (pregnant, chronic illnesses), causing serious staff shortages and thus restricting nurses’ abilities to conduct research. Likewise, the severity of the crisis situation made it difficult to return to respondents for further follow-up after the initial narratives had been submitted. This collection cannot be generalised as a comprehensive view of all oncology nurses across the Global South and may not reflect the experiences of all oncology nurses within a given institution or organisation.

## Conclusions

The COVID-19 pandemic is a truly global challenge and has set back hard-won progress in global health for years to come. Oncology nurses are at the centre of the cancer continuum of care, from public health and prevention through to survivorship or end of life; many transferable qualities they have used to help them handle the pandemic; ‘I am a nurse’.

The three research studies explicate: a) the psychological symptoms and their associated factors (e.g. geography and diagnosis) among patients with cancer in China and efforts to mitigate the consequences by a review of the early literature, b) cancer care during the pandemic in Brazil and the psychosocial impact on oncology nurses and c) the psychosocial aspects of care for patients being treated for cancer who are also infected with COVID-19 in a large cancer hospital in India. All three contributions note the serious negative psychosocial impact of the pandemic on both oncology nurses and their patients, yet acknowledge that the impact was not universal. Conducting research even during an unfolding pandemic is valuable to identify distinctions in psychosocial reactions and early interventions for the two groups, particularly in regard to accurate communication, caregiver support needs and oncology nurses’ overload from continuous frontline work demands.

The supplement (https://figshare.com/projects/Oncology_Nursing_in_the_Global_South_During_COVID-19_Supplement_to_Special_Issue_Ecancer_December_2021_/127586) of qualitative contributions eloquently illustrates the nurses’ sadnesses, vulnerabilities and indeed, strengths in the face of the pandemic. The nurses have brought to life the existing literature on the impact of COVID-19 on cancer care: interruptions to cancer care; resource shortages; poor mental wellbeing and critical staff shortages. They have also enriched our understanding of the impact of the pandemic on the nurses, their patients and their families: struggling with inappropriate and ineffective communication; their terror of catching the virus and dying, coping with social bullying and stigma and finding coping mechanisms, e.g., through humour, exercise and personal as well as professional accommodation.

Nurses, as ever, as critical members of any healthcare team, have risen to the challenge of delivering holistic care under duress, in hospital, outpatient and community settings during the pandemic. The eye-opening research and narratives of oncology nurses’ experiences in the Global South provide needed evidence of the psychosocial consequences and mitigation support (including liaising with local and international NGOs). This evidence will serve for strategic public health planning and health system realignment in preparation for the inevitable future pandemics.

## Conflicts of interest

The authors declare no conflicts of interest.

## Funding

No funding was received for this research.

## Figures and Tables

**Table 1.** Summary of all articles from Global South on the impact of COVID-19 in psychosocial aspects of oncology nursing and patients receiving cancer treatment. (See https://figshare.com/projects/Oncology_Nursing_in_the_Global_South_During_COVID-19_Supplement_to_Special_Issue_Ecancer_December_2021_/127586.)

Quantitative contributions: summaries					
Article Order	Country	Authors	Title	Methodology	Findings
1	India	Jagdish, P, D’Souza, A, Pawar, M, Goswami, S, & Patil, A	Assessment of psychosocial aspects of care for patients with cancer and COVID-19 at Tata Memorial hospital, India	Sample: 50 patients with cancer and COVID-19 recovering at home Access: phone interviews Tool: investigator designed psychosocial tool validated by seven experts	Need for additional psychosocial support for patients with cancer and their caregivers in India Oncology nurses overburdened Additional psychosocial professional and community resources essential
2	Brazil	Teixeira, TOA, Carvalho, LG, Camargo, GC & De Domenico, EBL	Cancer care in COVID-19 era and psychosocial impacts on oncology nursing in Brazil	Literature review Portuguese/Spanish literature (mainly from Brazil) Government reports on the impact of COVID-19 on oncology nursing care in Brazil	Reductions in screenings Delayed diagnosis Interruptions in treatment and follow-up Physical & psychological overload for nurses
3	China	Xu, B, Ng, MSN, & So, WKW	A review of psychological care of patients with cancer in mainland China during COVID-19	Literature review Psychological care of mainland China patients with cancer during COVID-19 pandemic All types of articles considered (e.g. empirical studies, discussion papers)	In oncology patients, high prevalence of psychological distress depression anxiety Lack of evidence on effective interventions for these psychological concerns

Supplement: qualitative nursing contributions: content focus identified according to four themes in reported literature review							
Article order	Country	Authors	Title	Interruptions in cancer care	Support/resource shortages & increased financial crisis	Staffing & nursing practice	Psychosocial impact on nurses and patients
1	Iran	Atashi, S. (Translator – Ahmadnia, S)	POEM ‘I am a nurse’				X
2	Wuhan China	Qian, S, Jie, L, Junshuai, L, YanLiu Rui, L, & Wanmin, Q	Experiences in Wuhan – ‘Battle against all the odds with hearts of love’			X	X
3	Shanghai China	He, M, Shen, N, Zhou, F, Cao, Q, & Sun, J	The psychosocial support for paediatric patients with cancer and nurses: experience from China	X	X	X	X
4	Latin America: Mexico, El Salvador, Peru	Spanish Interviewer Olarte-Sierra, MF Caballero, R (Mexico) Hernández, A (El Salvador) Celis, R (Peru)	Layers of care: Cancer Nursing in Latin America, amidst the COVID-19 pandemic		X X X		XX X X
5	Lebanon	Doumit, M	Healthcare Heroes: Lebanese Nurses experiences with COVID-19	X		X	X
6	Iraq	Anonymous author (Translator Abdullah, O)	Cancer caring during COVID-19 pandemic in Mosul	X	X	X	X
7	Ankara Turkey	Yılmaz P, Polat, S, & Yurduşen, S	Being a pediatric oncology nurse during COVID-Pandemic			X	X
8	Istanbul Turkey	Can, G, & Genç, Z	The Psychosocial Impact of COVID-19 Pandemic on Cancer Patients and Nurses In Turkey			X	X
9	Istanbul Turkey	Bingol, H, Karapınar, A Ekenel, M, & Kebudi, R	Challenges in caring for oncology patients during the COVID-19 pandemic in Istanbul, Turkey	X		X	X
10	Indonesia	Afiyanti, Y & Milanti, A	Psychosocial aspects of care of cancer patients during COVID-19 pandemic in Indonesia		X	X	X
11	Nigeria	Anarado, A, Opara, H, Mbadugha, C, Ulunma Obiora, M Millicent Ndikom, C, & Adebisi Hassan, M	COVID-19 And Psycho-Social Nursing Care Challenges Of Patients With Childhood Cancer And Their Families In Nigeria: A Nursing Perspective		X	X	X
12	Uganda	Ndagire, M & Nabakoosa, S	Delivering Pediatric Oncology Nursing Care Amidst COVID-19. Impact on nurses in Uganda		X	X	X
**Qualitative nursing contributions from NGOs: content focus identified according to four themes in reported literature review**							
13	Philippines	Buena Villarta, B & Auste, C	Nursing Care of Children with Cancer During the COVID-19 Pandemic: The Cancer Warriors Foundation, Inc. (CWFI) Experience	X	X		X
14	United Kingdom	Hollis, R & Cruise, M	‘Responding to the challenges faced by children, their families and health care professionals during COVID-19’	X	X	X	X
15	Zimbabwe	Tsikai, F	Nursing Care of Children with Cancer During the COVID-19 Pandemia: The Cancer Warriors Foundation, Inc. (CWFI) Experience		X	X	X

**Table 2.** Countries ranked by UN Development Programme HDI from lowest to highest.

Country	HDI^a^	Total population (2019)^b^	Total cancer cases in 2018^c^	% GDP spent on healthcare (2018)^d^
Nigeria	0.539	200,963,603	115,950	3.89
Uganda	0.544	44,269,587	32,617	6.53
Zimbabwe	0.571	14,645,473	17,465	4.73
India	0.645	1,366,417,756	1,157,294	3.54
El Salvador	0.673	6,453,550	10,326	7.11
Iraq	0.674	39,309,789	25,320	4.11
Philippines	0.718	108,116,622	141,021	4.40
Indonesia	0.718	270,625,567	348,809	2.87
Lebanon	0.744	6,855,709	17,294	8.35
China	0.761	1,441,860,295	4,285,033	5.35
Brazil	0.765	211,049,519	559,371	9.51
Peru	0.777	32,510,462	66,627	5.24
Mexico	0.779	127,575,529	190,667	5.37
Iran	0.783	82,913,893	110,115	8.66
Turkey	0.82	83,429,607	210,537	4.12
UK	0.932	67,530,161	446,942	10.00

a The health dimension is assessed by life expectancy at birth, the education dimension is measured by a mean of years of schooling for adults aged 25 years and more and expected years of schooling for children of school entering age. The standard of living dimension is measured by gross national income per capita http://hdr.undp.org/en/content/latest-human-development-index-ranking

b https://data.worldbank.org/indicator/SP.POP.TOTL

c Last year available, WHO cancer country profiles https://www.iccp-portal.org/map

d Last year available, https://data.worldbank.org/indicator/SH.XPD.CHEX.GD.ZS

## Endnote

1 Although HDI is another relevant way to organise countries, we chose to refer to the Global South. Not necessarily tied to geography, the critical concept Global South is used to include territories that have historically faced economic disadvantage and the consequences of colonial processes. Additionally, the Global South makes reference to territories that are geopolitically located in the periphery. As expressed by Dados and Connell, ‘the phrase “Global South” marks a shift from a focus on development or cultural difference toward an emphasis on geopolitical power relations’ [3, p.12].
